# Laparoscopic Sleeve-Fundoplication for Morbidly Obese Patients with Gastroesophageal Reflux: Systematic Review and Meta-analysis

**DOI:** 10.1007/s11695-020-05189-6

**Published:** 2021-01-03

**Authors:** Alberto Aiolfi, Giancarlo Micheletto, Jacopo Marin, Emanuele Rausa, Gianluca Bonitta, Davide Bona

**Affiliations:** 1 Department of Biomedical Science for Health, Division of General Surgery, Istitituto Clinico Sant’Ambrogio, University of Milan, Via Luigi Giuseppe Faravelli, 16, 20149 Milan, Italy; 2 Department of Pathophysiology and Transplantation, INCO and Department of General Surgery, Istituto Clinico Sant’Ambrogio, University of Milan, Milan, Italy

**Keywords:** Laparoscopic sleeve gastrectomy, Nissen fundoplication, Posterior fundoplication, Anterior fundoplication, GERD

## Abstract

**Introduction:**

Laparoscopic sleeve gastrectomy (LSG) has rapidly become popular with excellent results. However, LSG may exacerbate or increase the risk of “de novo” gastroesophageal reflux disease (GERD). Adding a fundoplication has been proposed to increase the lower esophageal sphincter competency. The aim of this study was to examine the current evidence and outcomes of sleeve-fundoplication (Sleeve-F).

**Materials and Methods:**

Systematic review and meta-analysis. Web of Science, PubMed, and Embase data sets were consulted.

**Results:**

Six studies (485 patients) met the inclusion criteria. The age of the patient population ranged from 17 to 72 years old and 82% were females. All patients underwent sleeve-fundoplication. Rossetti, Collis-Nissen, and Nissen were the most commonly performed fundoplications. The estimated pooled prevalence of postoperative leak, gastric perforation, and overall complications were 1.0% (95% CI = 0.0–2.0%), 2.9% (95% CI = 0.0–8.3%), and 9.8% (95% CI = 6.7–13.4%), respectively. The pooled reoperation rate was 4.1% (95% CI = 1.3–10%). There was no mortality. At 12-month follow-up, the estimated pooled BMI and %EWL were 29.9 kg/m^2^ (95% CI = 28.5–31.2) and 66.2% (95% CI = 59.3–71.1), respectively, while esophagitis, PPI consumption, and GERD rates were 8.0% (95% CI 3–21%), 7.8% (95% CI 5–13%), and 11% (95% CI 4–26%).

**Conclusions:**

This systematic review and meta-analysis shows that current evidence for Sleeve-F is limited with high postoperative gastric perforation and overall complication rates. Weight loss and GERD resolution seem promising in the short term; however, further studies are warranted to explore long-term effects with instrumental investigations. Sleeve-F should be considered cautiously while future well-structured randomized trials are warranted.

**Supplementary Information:**

The online version contains supplementary material available at 10.1007/s11695-020-05189-6.

## Introduction

Laparoscopic sleeve gastrectomy (LSG) has rapidly become popular worldwide for the treatment of morbid obesity because technically straightforward with excellent outcomes in terms of weight loss and comorbid resolution [1, 2]. Moreover, LSG is associated with reduced postoperative dumping syndrome, marginal ulcers, malabsorption, and internal hernia with improved quality of life [3, 4].

Despite the excellent long-term weight loss, LSG may exacerbate or increase the risk of “de novo” pathologic gastroesophageal reflux disease (GERD) and Barrett’s esophagus [5–7]. Crural repair has been described in an attempt to decrease the risk of GERD after LSG with conflicting results [8, 9]. Adding a fundoplication, with the intent of increase the lower esophageal sphincter (LES) competency, has been proposed recently; however, published studies are few while evidence is limited and puzzled.

The aim of this systematic review and meta-analysis was to examine the current evidence on the therapeutic role and outcomes of sleeve-fundoplication (Sleeve-F).

## Materials and Methods

### Search Strategy

A systematic review was performed according to the guidelines from the Preferred Reporting Items for Systematic Reviews and Meta-analyses (PRISMA) checklist [10] and Meta-analyses of Observation Studies in Epidemiology (https://www.editorialmanager.com/jognn/account/MOOSE.pdf). Institutional review board approval was not required. Literature search was conducted independently by three authors (AA, GB, JM) to identify the English-written published series on sleeve gastroplasty and fundoplication. Web of Science, PubMed, and Embase data sets were consulted matching the terms “sleeve gastrectomy,” “fundoplication,” “Nissen-sleeve,” and “N-sleeve” with “AND” and “OR.” The references of each article were assessed to complete the research [11].

### Inclusion and Exclusion Criteria

Inclusion criteria: (a) articles reporting outcomes for sleeve gastrectomy and fundoplication; (b) English written; (c) papers with the longest follow-up or the largest sample size in case of articles published by the same study group or based on the same data set. Exclusion criteria: (a) not English-written; (b) no clear methodology; (c) articles not reporting any of the a priori defined primary outcomes; (d) articles with less than 10 patients.

### Data Extraction

Three authors (AA, JM, GM) independently extracted data from eligible studies. Data extracted included study characteristics (first author name, year, and journal of publication), number of patients included in the series, time frame, clinical and demographic characteristics of patients’ population, type of surgical procedure, and postoperative outcomes. Disagreements between authors were resolved by consensus; if no agreement could be reached, a fourth senior author (DB) made the decision.

### Quality Assessment

Three investigators (AA, GB, GL) independently assessed the methodological quality of the enrolled papers using the Newcastle-Ottawa Scale (NOS) [12]. Each study is judged on a “star system” based on the selection of the study groups and the ascertainment of outcome of interest. Each study could earn a maximum of 9 stars. Studies with low quality score (NOS < 6) were excluded.

### Outcomes

Primary outcomes: postoperative leak, perforations, and overall complication rate. Secondary outcomes: bleeding, reoperation, operative time (minutes), hospital length of stay (days), body mass index (BMI), percentage excess weight loss (%EWL), esophagitis, PPI use, and incidence of clinical GERD at a minimum 12-month follow-up. GERD was defined, according to the Montreal’s definition, as a condition that develops when the reflux of stomach contents causes troublesome symptoms and/or complications [13].

### Statistical Analysis

We performed a random effect Frequentist meta-analysis. Binary outcomes were pooled using generalized linear mixed models with logit transformation [14, 15]. The maximum-likelihood estimator was used to estimate the between-study variance (*τ*^2^) and the non-parametric bootstrap was used to calculate its bias-corrected and 95% confidence interval. The inverse-variance random effects meta-analysis was performed by conventional methods using the DerSimonian-Laird estimator for estimate between-study variance (*τ*^2^) was performed [16, 17]. Clopper-Pearson 95% confidence intervals for individual were computed [18]. Statistical heterogeneity was evaluated (*I*^2^ index): value of 25% or smaller was defined as low heterogeneity, value between 50 and 75% as moderate heterogeneity, and 75% or larger as high heterogeneity [19, 20]. Small study and publication bias effects were assessed by trim and fill funnel plot visual inspection and Egger tests [21, 22]. Prediction interval for treatment effect of a new study is calculated according to Borestein [23]. As sample size is not the same in all studies, we gradually removed small sample size to perform a sensitivity analysis to assess stability of results. Two-sided *p* values were considered statistically significant when < 0.05. All analyses and graphical representations were carried out using R version 3.2.2 software [24].

## Results

### Systematic Review

**Fig. 1:**
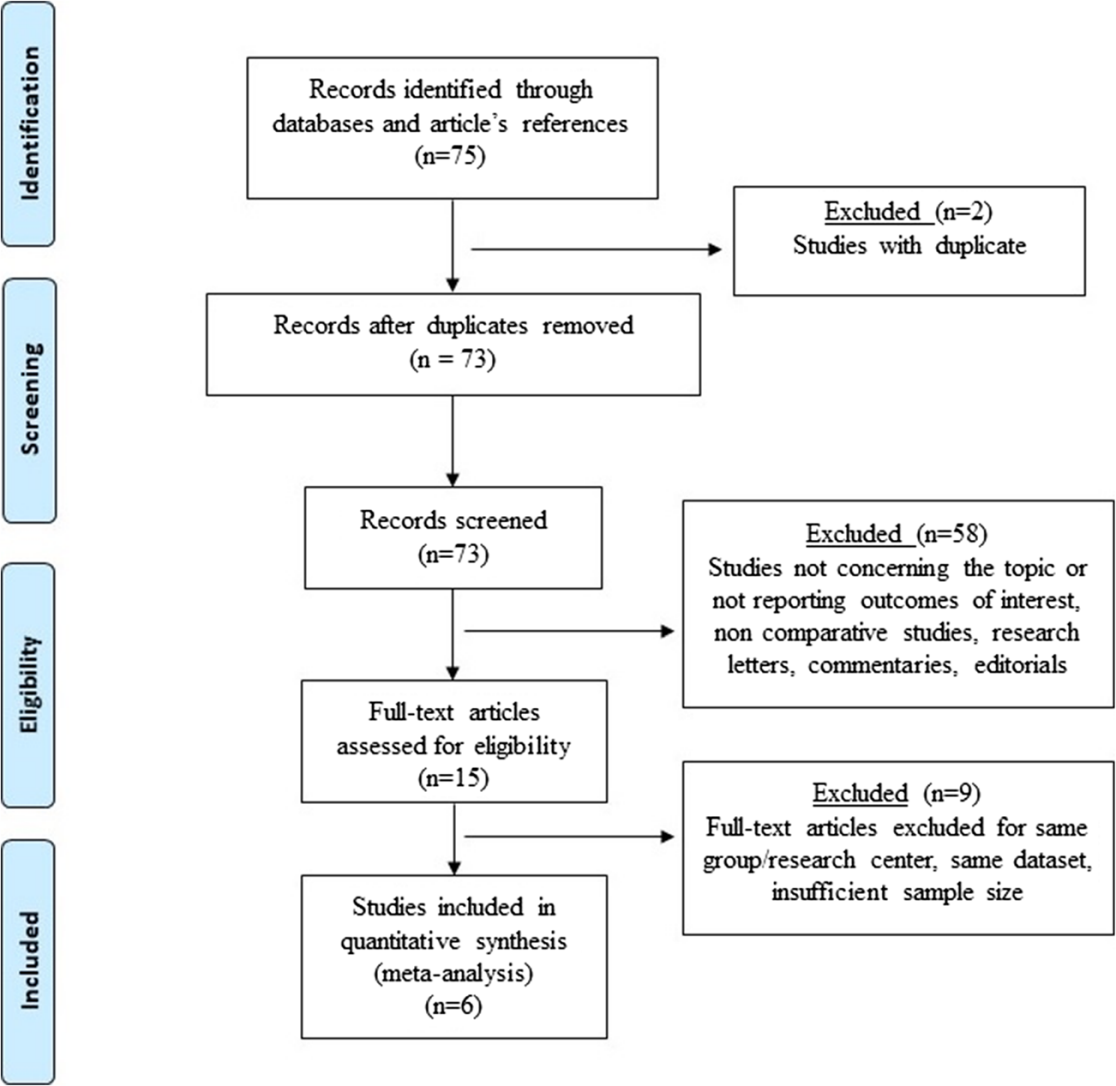
The Preferred Reporting Items for Systematic Reviews and Meta-analyses (PRISMA) checklist diagram

Six studies published between 2015 and 2020 met the inclusion criteria (Fig. 1). The total number of patients was 485; the sample size of the individual studies ranged from 15 to 220. All reports were observational, cohort studies; each study earned a NOS score of 7 or 8 (median 7.3), suggesting a fair quality level. Demographic, clinical, and operative variables of the patient sample are shown in Table 1. Three papers included more than 50 patients. The age of the included patients ranged from 17 to 72 years old and the majority were females (81.8%). Patients’ comorbidities were reported in five articles (415 patients) while the American Society of Anesthesiologists (ASA) physical status classification was not reported in any of the included articles. Reported comorbidities were hypertension (61.7%), non-alcoholic fatty liver disease (47%), hyperlipemia (31.8%), obstructive sleep apnea syndrome (26.5%), and type II diabetes (21%). The BMI before Sleeve-F ranged from 31 to 69 kg/m^2^. The indication for Sleeve-F was morbid obesity with concomitant clinical GERD (92.1%) or morbid obesity. Preoperative clinical definition of GERD was according to the Montreal definition.

**Table 1:** Demographics and clinical data of 485 patients undergoing sleeve-fundoplication. Values are reported as mean ± standard deviation, median (range), and number (percentage). BMI Body Mass Index. HLOS hospital length of stay. NOS: Newcastle-Ottawa Scale

Author, year	No. patients	Age (years)	Females no.	BMI (kg/m^2^)	Surgical procedure	Bougie size	Operative time (min)	HLOS (days)	Follow-up (months)	NOS score
Da Silva et al., 2015 [25]	122	42.4 (18–72)	119	42.5 (31–69)	Sleeve-Collis-Nissen (hiatoplasty)	36Fr	91 (75–120)	2 (1–3)	36	7
Moon et al., 2016 [26]	33	49.9 (29–63)	28	42.8 (33–58)	Sleeve-anterior fundoplication (hiatoplasty)	34Fr	100.9 (67–146)	1.9 (1–13)	12	8
Nocca et al., 2016 [27]	25	41 (20–65)	13	42 (35–53)	Sleeve-Nissen (hiatoplasty)	37Fr	84 (54–106)	5 (4–10)	12	8
Lasnibat et al., 2017 [28]	15	46.2	14	33.9 ± 2.1	Sleeve-Nissen (hiatoplasty)	34Fr	157 ± 22	4.6	12	7
Amor et al., 2020 [29]	70	42 (17–70)	56	40 (35–60)	Sleeve-Nissen (hiatoplasty)	37Fr	62 (30–120)	2 (2–3)	12	8
Olmi et al., 2020 [30]	220	42.7 (18–67)	167	42.6 (31–63)	Sleeve-Rossetti (hiatoplasty *n* = 4)	38 Fr	48 (20–120)	4.2 (3–46)	24	8

All patients underwent laparoscopic sleeve gastrectomy with concomitant fundoplication (Sleeve-F). Overall, 452 (93.2%) underwent posterior fundoplication while 33 (6.8%) underwent anterior fundoplication. The most commonly performed posterior fundoplication was Rossetti fundoplication (220 patients) followed by Collis-Nissen fundoplication (*n* = 122), and Nissen fundoplication (*n* = 110). Different Bougie sizes were used according to operating surgeons’ preference to calibrate the sleeve gastroplasty. There was only one conversion to open surgery because of bleeding. The operative time ranged from 30 to 146 min; 269 patients underwent concomitant posterior cruroplasty while 28 patients underwent cholecystectomy. Gastric perforation (2.5%), bleeding (2.1%), stenosis (1.2%), and pulmonary complications (1%) were the most commonly reported complications. There was no mortality. Cost analysis and postoperative quality of life evaluation were not reported in any of the included studies.

### Meta-analysis

#### Primary Outcomes

**Fig. 2:**
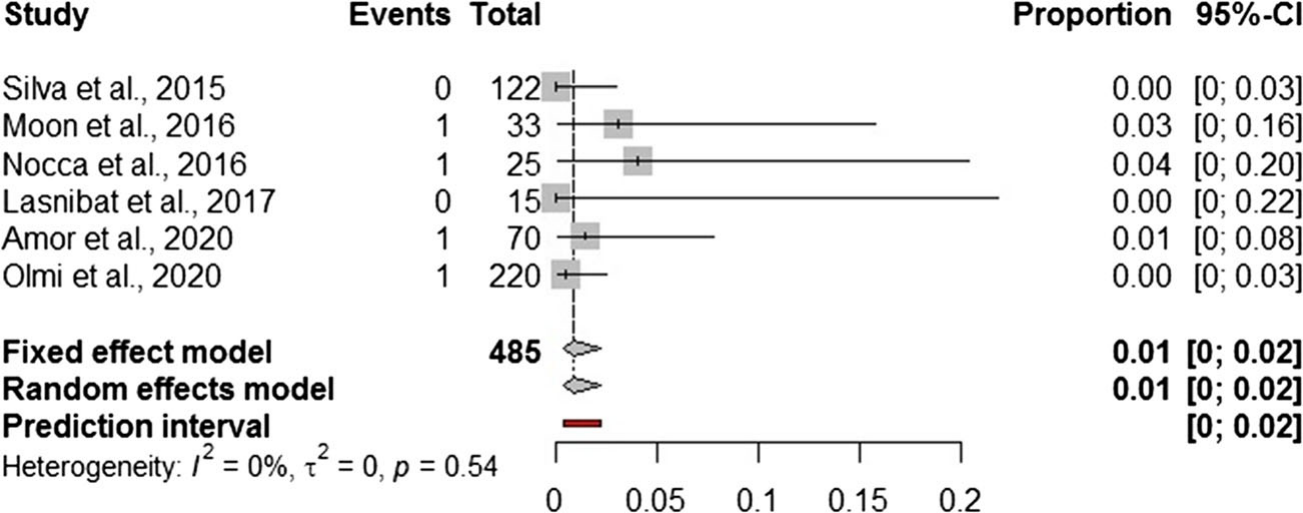
Forest plot of postoperative leak

**Fig. 3:**
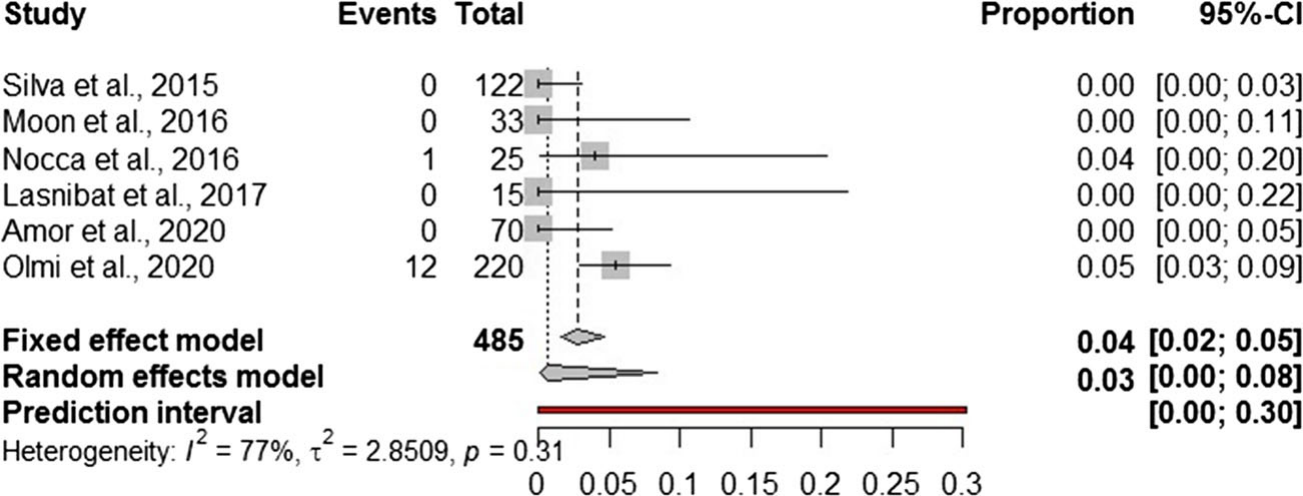
Forest plot of postoperative gastric perforation

**Fig. 4:**
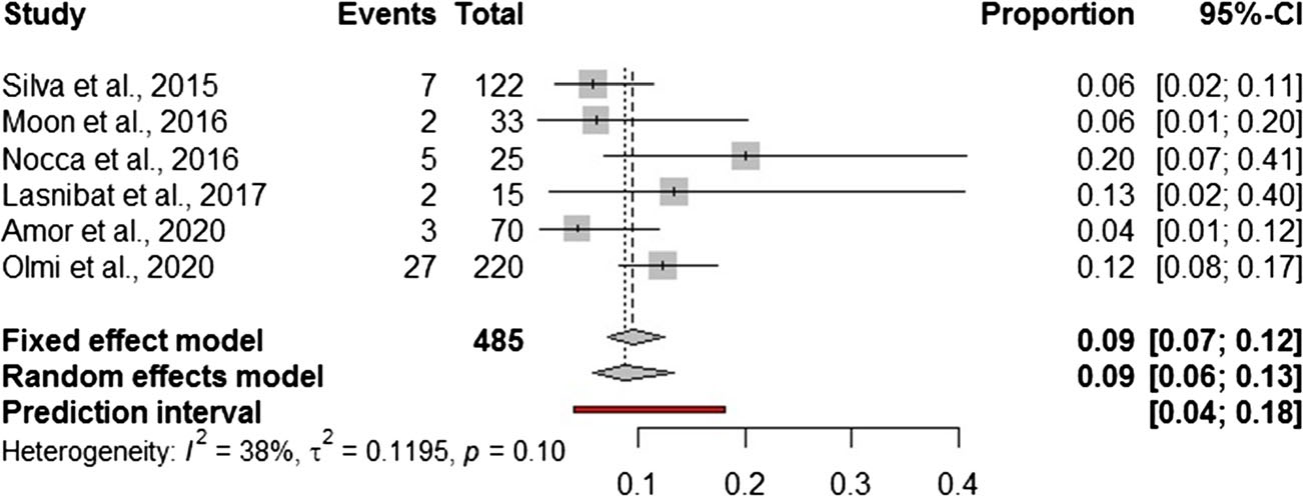
Forest plot of postoperative overall complications

In addition to a systematic review, we performed a Frequentist meta-analysis. Considering a random effect model, the estimated pooled prevalence of postoperative leak (6 studies, 485 patients) is 1.0% (95% CI = 0.0–2.0%) (Fig. 2). The prediction lower and upper limits are 0.0% and 2.0%, respectively. The heterogeneity index is zero (*I*^2^ = 0.0%, 95% CI = 0.0–9.7%; *p* = 0.54). The sensitivity analysis shows the robustness of results (Supplementary Table 1). The estimated pooled prevalence of gastric valve perforation (6 studies, 485 patients) is 2.9% (95% CI = 0.0–8.3%) (Fig. 3). The prediction lower and upper limits are 0.0% and 30.0%, respectively. The heterogeneity index is high (*I*^2^ = 76.5%, 95% CI = 53.2–92.4%; *p* = 0.31). The one-leave out sensitivity analysis shows that the pooled prevalence could increase up to 4.5–5% with a decrease of related heterogeneity (up to *I*^2^ = 23.0%) (Supplementary Table 1). The estimated pooled prevalence of overall complications (6 studies, 485 patients) is 9.8% (95% CI = 6.7–13.4%) (Fig. 4). The prediction lower and upper limits are 4.1% and 18.3%, respectively. The heterogeneity index is moderate (*I*^2^ = 38%, 95% CI = 24.2–60.7%; *p* = 0.10). The sensitivity analysis shows the robustness of point estimation and 95% CI (Supplementary Table 1).

#### Secondary Outcomes

The estimated pooled mean operative time (6 studies, 485 patients) and hospital length (6 studies, 485 patients) of stay are 90 min (95% CI = 68.6–111.3 *I*^2^ = 100%) and 2.95 days (95% CI = 5.6–3.3). The heterogeneity index is high (*I*^2^ = 95% and 97%, respectively). The estimated pooled prevalence of reoperation (5 studies, 363 patients) is 4.0% (95% CI = 1.0–10.0%) with a low heterogeneity (*I*^2^ = 21%). The estimated incidences of postoperative esophagitis and PPI consumption at a minimum of 12-month follow-up are 8.0% (3–21%) and 7.8% (5–13%), respectively, with a moderate related heterogeneity (*I*^2^ = 46% and 48%, respectively). The postoperative estimated pooled BMI (6 studies, 363 patients) and %EWL (6 studies, 357) at a minimum of 12-month follow-up are 29.9 kg/m^2^ (95% CI = 28.5–31.2) and 66.2% (95% CI = 59.3–71.1). The related heterogeneity index is high (*I*^2^ = 71.9%, *p* < 0.01). The sensitivity analysis for operative time and hospital length of stay show the robustness of the results. The sensitivity analysis for %EWL shows that by omitting the study by Antonopulos et al., the heterogeneity decreases to low (31.7%). All secondary outcomes are reported in Table 2.

**Table 2:** Secondary outcomes. Values are expressed as pooled proportions and 95% confidence intervals (95% CI). *I*^2^: heterogeneity. *BMI* body mass index. *HLOS* hospital length of stay. *%EWL* percentage excess weight loss. *PPI* proton pump inhibitors. *GERD* gastroesophageal reflux

Outcomes	Proportion (95% CI)	*I*^2^
Postoperative bleeding	2.0% (1.1–4.3%)	0.0%
Reoperation	4.1% (1.3–10%)	21%
Operative time (minutes)	90 (68.7–111.3)	95%
HLOS (days)	2.95 (2.57–3.32)	97%
BMI (kg/m^2^)	30.1 (28.8–31.35)	96%
%EWL	64.4 (58.9–69.9)	97%
Esophagitis	8% (3–21%)	46%
Post-op PPI use	7.8% (5–13%)	48%
Post-op GERD	11% (4–26%)	90%

## Discussion

This systematic review and meta-analysis shows that literature evidence reporting data for Sleeve-F is lacking and supported by retrospective observational studies. According to current data, Sleeve-F seems feasible and safe with acceptable postoperative leak rate, bleeding, and mortality while gastric perforation, reoperations, and overall complications are noteworthy. While instrumental postoperative GERD evaluation is lacking, the effectiveness of Sleeve-F up to 1-year follow-up seems promising with decreased BMI and %EWL.

LSG is considered a technically straightforward procedure while the entire removal of the gastric fundus with the visualization of the left diaphragm crus is a technical key point [31, 32]. Concerns about postoperative GERD have been risen with a reported incidence up to 25–30% of patients [1, 2]. While a careful preoperative patients’ selection is mandatory, several factors may be implicated in the exacerbation or “de novo” development of postoperative GERD [33]. Decreased gastric emptying, lower LES pressure, blunting the His angle, partial section of the muscular Helvetius collar, decreased gastric compliance/volume, and increased gastric pressure have been advocated as possible influencing factors [29, 34, 35]. The choice of the most suitable weight-loss procedure should be carefully evaluated especially in patients with a pre-existing clinical or latent GERD. Many surgeons are reluctant to offer LSG in patients with GERD that are offered LRYGB while other surgeons support the choice of LSG. Furthermore, morbidly obese patients with GERD that refused RYGB represent a challenge [25, 36, 37].

Our systematic review and meta-analysis showed that Sleeve-F seems technically feasible and safe. There was no mortality in the patient population and the incidence of postoperative leak and bleeding was 1.0% (95% CI = 0.0–2.0%) and 2.0% (95% CI = 1.1–4.3%). The related heterogeneity was 0.0% and the sensitivity analysis added robustness to the result. The wrapping of the His angle with the antireflux valve has been proposed as a possible protective factor with a reduced leak risk [30]. The rationale is to cover the His angle moving the staple line to a better vascularized area [27]. Notably, the estimated pooled prevalence of postoperative leak seems equivalent to other studies reporting outcomes for standard LSG [1, 2, 38]. The pooled gastric perforation rate was 2.9%. Notably, the upper 95% CI limit was 8.3% and related heterogeneity was high (*I*^2^ = 76.5%). The sensitivity analysis showed that the one-leave out study omission determined an increase in the incidence of gastric perforation (4.5–5%) with a decrease in related heterogeneity to low values (up to *I*^2^ = 23%). Therefore, we believe that this pooled rate is more reliable and statistically robust. The postoperative gastric perforation is an event that is totally different from leak, that is the reason why we performed two different quantitative analyses. Different theories have been risen ranging from incongruous manipulation of the gastric fundus during the operation, incorrect grasper handling, thermic injury, to inadequate gastric valve vascularization even in the presence of large intramural vessels and gastric valve perfusion at the intraoperative green indocyanine test [27, 30]. Caution is mandatory while interpreting this outcome because of potentially being influenced by diverse surgical techniques, surgeons’ experience, valve anatomy, outcomes reporting, definition of postoperative complications, preoperative comorbidities, and patients’ selection bias. The pooled reoperation and overall complication rate were 4.0% and 9.8%, respectively, with a low-moderate heterogeneity. The most commonly reported cause of reoperation was perforation of the gastric valve; laparoscopic revision consisted in resection of the gastric valve, perigastric abscess drainage, and conversion to a standard LSG in the majority of cases. The overall complication rate is higher compared to other series describing outcomes for LSG [1, 2, 39, 40]. This may be influenced by the effect of gastric perforations with perigastric collection that contributed to a substantial increase in the overall complication rate. These results should be considered cautiously because of possibly being influenced by the initial learning curve phase, in a novel, non-standardized, and experimental technique.

The pooled mean operative time and hospital length of stay were 90 min (95% CI = 68.7–111.3) and 2.95 days (95% CI = 2.6–3.3) with high-related heterogeneity (95% and 97%, respectively). This may be explained by several factors such as patients’ age, comorbidities, preoperative BMI, surgical technique, valve anatomy, need for hiatal hernia repair, concomitant cholecystectomy, hospital volume, presence of peritoneal adhesions, and surgeons’ expertise. The mean pooled BMI and %EWL at 1-year follow-up were 30.1 kg/m^2^ (95% CI = 28.8–31.3) and 64.4% (95% CI = 58.9–69.9), respectively, with a high-related heterogeneity (> 90%). These results seem comparable to BMI and %EWL at 1-year follow-up after standard LSG [1, 2, 41, 42]. Again, caution is mandatory because of possible confounders related to compliance with dietary regimens, different bougie size, and limited follow-up that do not allow to draw conclusive and robust evidence. Furthermore, the purpose of leaving a small portion of gastric fundus could compromise the weight-loss effect with a possible criticism for weight-regain [43, 44]. In an attempt to explore medium-term follow-up data, Olmi and colleagues reported data for 58 patients that concluded the 2-year follow-up analysis. The reported BMI and %EWL were 27.8 and 74.4%, respectively [30]. In another study by da Silva et al., 33 patients were followed up and completed the 3-year postoperative evaluation. The authors reported a %EWL of 60.4 ± 8.1% with a significant decrease in postoperative esophagitis (100 vs. 13.6%) and PPI consumption (92 vs. 13.6%) compared to preoperative evaluation [25].

Furthermore, it was difficult to assess the effect of Sleeve-F on PPI consumption, esophagitis, and clinical GERD because data were reported as aggregated and because of the lack of individual patient data trajectory. Except Olmi and colleagues, all included studies reported data for morbidly obese patients with a preoperative GERD that was reported as improved in the follow-up. Preoperative esophagitis and PPI consumption were reported in 55.7% and 83% of patients, respectively. Pooled data showed an incidence of postoperative esophagitis, PPI consumption, and GERD of 8% (95% CI = 3–21%), 7.8% (95% CI = 5–13%), and 11% (95% CI = 4–26%), respectively. While related heterogeneity for esophagitis and PPI consumption was moderate, a high-related heterogeneity was found for clinical GERD. This may be attributable to the clinical and endoscopic definition of GERD according to the Montreal classification in combination with patients’ reporting. Specifically, the correlation between symptoms and esophagitis is not a sensitive marker for pathologic GERD while heartburn may be referred by some patients with esophageal hypersensitivity or functional disorders that are not sustained by a true pathologic reflux [29, 45]. Therefore, these data are prone to criticism and, in the future, it would be desirable to obtain more robust evidence by objective data assessment with pH-impedance 24-h study or Bravo pH test evaluation in combination with esophageal manometry [46].

Lastly, the choice of fundoplication was left to surgeons’ preference. Olmi and colleagues adopted the modified Rossetti fundoplication because of the limited esophageal and crura dissection with a reduced need for posterior hiatoplasty (only 4 patients). The authors reported the creation of a small retroesophageal window for the passage of the fundus without leaving a wide space. Furthermore, the fundoplication was fashioned with only gastro-gastric stitches and not sutured to the esophagus to avoid vagal nerve injuries and prevent gastric emptying disorders [30]. Other authors described a Nissen-sleeve fundoplication with a more extended esophageal dissection in the posterior mediastinum to obtain at least 5 cm of intra-abdominal esophagus. The short Nissen valve (2.5–3 cm) was fixed anteriorly at the esophagus and laterally to the right diaphragmatic pillar after the closure of the hiatus. On the other hand, Moon et al. described the fashioning of an anterior 120° fundoplication, sutured to the right and left pillars, after having performed a minimal diaphragmatic dissection. The authors justify their choice because of the fear of leaving too much gastric fundus that would have been affecting the weight-loss effect [26]. Notably, the choice of the type of fundoplication may influence outcomes and should be considered as a possible source of selection bias and heterogeneity. Therefore, evidence to support one fundoplication over another is lacking and future studies should focus on this comparison.

We acknowledge that this review does have some limitations related to possible publication bias due to exclusion of non-English articles, heterogeneity of some of the studies included, and retrospective nature of the included series. In addition, the reason for why each patient had a specific surgical approach with different valve anatomy was based on surgeon preference and may represent some selection bias and source of heterogeneity. Finally, the limited patient cohort may constitute a further limitation. However, it should be noted that Sleeve-F is a relatively new procedure with few published studies and limited patients’ cohorts. Up to our knowledge, this is the first meta-analysis providing quantitative data on Sleeve-F. Though, all the studies currently available supporting this surgery are few and observational. Therefore, this meta-analysis also aims to plea for further qualitative and standardized studies in order to codify the surgical procedure and better assess postoperative outcomes.

## Conclusions

This systematic review and meta-analysis shows that current evidence for Sleeve-F is limited with high postoperative gastric perforation and overall complication rates. The effectiveness of Sleeve-F in terms of weight loss, GERD resolution, esophagitis remission, and PPI suspension seems promising in the short term but further studies are warranted to explore its effect in the medium-long term with objective instrumental investigations. Sleeve-F should be considered cautiously while future well-structured randomized trials are warranted.

## Supplementary Information

ESM 1 (DOCX 13 kb)

## References

[1] Gagner M, Deitel M, Erickson AL, Crosby RD (2013). Survey on laparoscopic sleeve gastrectomy (LSG) at the Fourth International Consensus Summit on Sleeve Gastrectomy. Obes Surg.

[2] Gagner M, Hutchinson C, Rosenthal R (2016). Fifth International Consensus Conference: current status of sleeve gastrectomy. Surg Obes Relat Dis.

[3] Nedelcu M, Noel P, Iannelli A, Gagner M (2015). Revised sleeve gastrectomy (re-sleeve). Surg Obes Relat Dis.

[4] Porta A, Aiolfi A, Musolino C, Antonini I, Zappa MA (2017). Prospective comparison and quality of life for single-incision and conventional laparoscopic sleeve gastrectomy in a series of morbidly obese patients. Obes Surg.

[5] Braghetto I, Korn O. Late esophagogastric anatomic and functional changes after sleeve gastrectomy and its clinical consequences with regards to gastroesophageal reflux disease. Dis Esophagus. 2019 Jun 1;32(6):doz020. 10.1093/dote/doz020 31076757

[6] Sebastianelli L, Benois M, Vanbiervliet G, Bailly L, Robert M, Turrin N, Gizard E, Foletto M, Bisello M, Albanese A, Santonicola A, Iovino P, Piche T, Angrisani L, Turchi L, Schiavo L, Iannelli A (2019). Systematic endoscopy 5 years after sleeve gastrectomy results in a high rate of Barrett’s esophagus: results of a multicenter study. Obes Surg.

[7] Sharma A, Aggarwal S, Ahuja V, Bal C (2014). Evaluation of gastroesophageal reflux before and after sleeve gastrectomy using symptom scoring, scintigraphy, and endoscopy. Surg Obes Relat Dis.

[8] Snyder B, Wilson E, Wilson T, Mehta S, Bajwa K, Klein C (2016). A randomized trial comparing reflux symptoms in sleeve gastrectomy patients with or without hiatal hernia repair. Surg Obes Relat Dis.

[9] Soricelli E, Iossa A, Casella G, Abbatini F, Calì B, Basso N (2013). Sleeve gastrectomy and crural repair in obese patients with gastroesophageal reflux disease and/or hiatal hernia. Surg Obes Relat Dis.

[10] Moher D, Liberati A, Tetzlaff J, et al. Preferred reporting items for systematic reviews and meta-analyses: the PRISMA statement. PLoS Med 2009 21;6(7):e1000097. 10.1371/journal.pmed.1000097 PMC2707599 19621072

[11] Goossen K, Tenckhoff S, Probst P, Grummich K, Mihaljevic AL, Büchler MW, Diener MK (2018). Optimal literature search for systematic reviews in surgery. Langenbeck’s Arch Surg.

[12] Stang A (2010). Critical evaluation of the Newcastle-Ottawa scale for the assessment of the quality of nonrandomized studies in meta-analyses. Eur J Epidemiol.

[13] Vakil N, van Zanten SV, Kahrilas P, Global Consensus Group (2006). The Montreal definition and classification of gastroesophageal reflux disease: a global evidence-based consensus. Am J Gastroenterol.

[14] Lin L, Chu H (2020). Meta-analysis of proportions using generalized linear mixed models. Epidemiology..

[15] Schwarzer G, Chemaitelly H, Abu-Raddad LJ, Rücker G (2019). Seriously misleading results using inverse of Freeman-Tukey double arcsine transformation in meta-analysis of single proportions. Res Synth Methods.

[16] DerSimonian R, Laird N (1986). Meta-analysis in clinical trials. Control Clin Trials.

[17] Ferrari D, Aiolfi A, Bonitta G, Riva CG, Rausa E, Siboni S, et. al. (2018) Flexible versus rigid endoscopy in the management of esophageal foreign body impaction: systematic review and meta-analysis. World Journal of Emergency Surgery 13(1). 10.1186/s13017-018-0203-4 PMC6134522 30214470

[18] Clopper CJ, Pearson ES (1934). The use of confidence or fiducial limits illustrated in the case of the binomial. Biometrika.

[19] Aiolfi A, Asti E, Rausa E, Bernardi D, Bonitta G, Bonavina L (2018). Trans-gastric ERCP after Roux-en-Y gastric bypass: systematic review and meta-analysis. Obes Surg.

[20] Higgins JP, Thompson SG (2002). Quantifying heterogeneity in a meta-analysis. Stat Med.

[21] Anzures-Cabrera J, Higgins JP (2010). Graphical displays for meta-analysis: an overview with suggestions for practice. Res Synth Methods.

[22] Egger M, Davey Smith G, Schneider M et al (1997) Bias in meta-analysis detected by a simple, graphical test. BMJ 13;315(7109):629-634 10.1136/bmj.315.7109.629 PMC2127453 9310563

[23] Borenstein M, Hedges LV, Higgins JPT, Rothstein HR (2009) Introduction to meta-analysis, John Wiley & Sons, Ltd, Chichester, UK

[24] R Development Core Team (2015) A language and environment for statistical computing. R Foundation for Statistical Computing, Vienna, Austria. ISBN 3-900051-07-0.

[25] da Silva LE, Alves MM, El-Ajouz TK (2015). Laparoscopic Sleeve-Collis-Nissen gastroplasty: a safe alternative for morbidly obese patients with gastroesophageal reflux disease. Obes Surg.

[26] Moon RC, Teixeira AF, Jawad MA (2017). Safety and effectiveness of anterior fundoplication sleeve gastrectomy in patients with severe reflux. Surg Obes Relat Dis.

[27] Nocca D, Skalli EM, Boulay E, Nedelcu M, Michel Fabre J, Loureiro M (2016). Nissen Sleeve (N-Sleeve) operation: preliminary results of a pilot study. Surg Obes Relat Dis.

[28] Lasnibat JP, Braghetto I, Gutierrez L (2017). Sleeve gastrectomy and fundoplication as a single procedure in patients with obesity and gastroesophageal reflux. Arq Bras Cir Dig.

[29] Amor IB, Casanova V, Vanbiervliet G, Bereder JM, Habitan R, Kassir R, Gugenheim J (2020). The Nissen-Sleeve (N-Sleeve): results of a cohort study. Obes Surg.

[30] Olmi S, Uccelli M, Cesana GC, Ciccarese F, Oldani A, Giorgi R, de Carli SM, Villa R (2020). Modified laparoscopic sleeve gastrectomy with Rossetti antireflux fundoplication: results after 220 procedures with 24-month follow-up. Surg Obes Relat Dis.

[31] Gagner M, Ramos A, Palermo M, et al. The perfect sleeve gastrectomy. A clinical guide to evaluation, treatment, and techniques. Cham, Switzerland: Springer, 2020.

[32] Palermo M, Edgardo S. Laparoscopic sleeve gastrectomy: how I do it J Laparoendosc Adv Surg Tech 2019, 10.1089/lap.2019.0452. 10.1089/lap.2019.0452 31364907

[33] Patti MG, Schlottmann F (2018). Gastroesophageal reflux after sleeve gastrectomy. JAMA Surg.

[34] Mahawar KK, Jennings N, Balupuri S, Small PK (2013). Sleeve gastrectomy and gastro-oesophageal reflux disease: a complex relationship. Obes Surg.

[35] Samakar K, McKenzie TJ, Tavakkoli A (2016). The effect of laparoscopic sleeve gastrectomy with concomitant hiatal hernia repair on gastroesophageal reflux disease in the morbidly obese. Obes Surg.

[36] Nocca D, Nedelcu M, Loureiro M, Palermo M, Silvestri M, Jong A, Ramos A (2020). The Nissen sleeve gastrectomy: technical considerations. J Laparoendosc Adv Surg Tech A.

[37] Rebecchi F, Allaix ME, Patti MG, Schlottmann F, Morino M (2017). Gastroesophageal reflux disease and morbid obesity: to sleeve or not to sleeve?. World J Gastroenterol.

[38] Rosenthal RJ, Diaz AA, Arvidsson D (2012). International sleeve gastrectomy expert panel consensus statement: best practice guidelines based on experience of > 12,000 cases. Surg Obes Relat Dis.

[39] Kichler K, Rosenthal RJ, DeMaria E, Higa K (2019). Reoperative surgery for nonresponders and complicated sleeve gastrectomy operations in patients with severe obesity. An international expert panel consensus statement to define best practice guidelines. Surg Obes Relat Dis.

[40] Angrisani L, Santonicola A, Iovino P, Vitiello A, Higa K, Himpens J, Buchwald H, Scopinaro N (2018). IFSO worldwide survey 2016: primary, endoluminal, and revisional procedures. Obes Surg.

[41] Cottam S, Cottam D, Cottam A (2019). Sleeve gastrectomy weight loss and the preoperative and postoperative predictors: a systematic review. Obes Surg.

[42] Peterli R, Wölnerhanssen BK, Peters T, Vetter D, Kröll D, Borbély Y, Schultes B, Beglinger C, Drewe J, Schiesser M, Nett P, Bueter M (2018). Effect of laparoscopic sleeve gastrectomy vs laparoscopic Roux-en-Y gastric bypass on weight loss in patients with morbid obesity: the SM-BOSS randomized clinical trial. JAMA..

[43] Arman GA, Himpens J, Dhaenens J, Ballet T, Vilallonga R, Leman G (2016). Long-term (11+years) outcomes in weight, patient satisfaction, comorbidities, and gastroesophageal reflux treatment after laparoscopic sleeve gastrectomy. Surg Obes Relat Dis.

[44] Himpens J, Dobbeleir J, Peeters G (2010). Long-term results of laparoscopic sleeve gastrectomy for obesity. Ann Surg.

[45] Lim CH, Lee PC, Lim E, Tan J, Chan WH, Tan HC, Ganguly S, Tham KW, Eng A (2019). Correlation between symptomatic gastro-esophageal reflux disease (GERD) and erosive esophagitis (EE) post-vertical sleeve gastrectomy (VSG). Obes Surg.

[46] Del Genio G, Tolone S, Gambardella C (2020). Sleeve gastrectomy and anterior fundoplication (D-SLEEVE) prevents gastroesophageal reflux in symptomatic GERD. Obes Surg.

