# Correction to: Laparoscopic Sleeve-Fundoplication for Morbidly Obese Patients with Gastroesophageal Reflux: Systematic Review and Meta-analysis

**DOI:** 10.1007/s11695-022-06105-w

**Published:** 2022-05-16

**Authors:** Alberto Aiolfi, Giancarlo Micheletto, Jacopo Marin, Emanuele Rausa, Gianluca Bonitta, Davide Bona

**Affiliations:** 1 Department of Biomedical Science for Health, Division of General Surgery, Istitituto Clinico Sant’Ambrogio, University of Milan, Via Luigi Giuseppe Faravelli, 16, 20149 Milan, Italy; 2 Department of Pathophysiology and Transplantation, INCO and Department of General Surgery, Istituto Clinico Sant’Ambrogio, University of Milan, Milan, Italy

**Correction to: Obesity Surgery (2021) 31:1714-1721**

10.1007/s11695-020-05189-6

The article Laparoscopic Sleeve-Fundoplication for Morbidly Obese Patients with Gastroesophageal Reflux: Systematic Review and Meta-analysis, written by Alberto Aiolfi, Giancarlo Micheletto, Jacopo Marin, Emanuele Rausa, Gianluca Bonitta, and Davide Bona, was originally published online on the publisher’s internet portal on January 3, 2022, with open access under a Creative Commons Attribution (CC BY) license 4.0. With the authors‘ decision to cancel open access, the copyright of the article changed on May 11, 2022, to © The Author(s), under exclusive licence to Springer Science+Business Media, LLC part of Springer Nature 2022 with all rights reserved.

