# Supplementary material for: Laparoscopic Sleeve-Fundoplication for Morbidly Obese Patients with Gastroesophageal Reflux: Systematic Review and Meta-analysis
Source: Obes Surg. 2021 Jan 3;31(4):1714–21. doi: 10.1007/s11695-020-05189-6 (PMC8012327; doi:10.1007/s11695-020-05189-6)
Supplement: Supplementary file 1 — (DOCX 13 kb) [file 11695_2020_5189_MOESM1_ESM.docx]

| L**eak** | Proportion (95% CI) | I^2^ |
| --- | --- | --- |
| Omitting Silva et al., 2015 | 1.79 (0.0-4.4) | 0.0 |
| Omitting Moon et al., 2016 | 1.3 (0.0-3.4) | 0.0 |
| Omitting Nocca et al., 2016 | 1.2 (0.0-3.2) | 0.0 |
| Omitting Lasnibat et al., 2017 | 1.4 (0.0-3.5) | 0.0 |
| Omitting Amor et al., 2020 | 1.6 (0.0-4.1) | 1.1 |
| Omitting Olmi et al., 2020 | 2.1 (0.0-5.45) | 0.0 |
|  |  |  |
| **Pooled estimate** | **1.0 (0.0-2.2)** | **0.0** |
|  |  |  |
| **Gastric perforation** | Proportion (95% CI) | I^2^ |
| Omitting Silva et al., 2015 | 4.65 (2.8-7.65) | 0.0 |
| Omitting Moon et al., 2016 | 4.96 (2.7-7.3) | 23.1 |
| Omitting Nocca et al., 2016 | 4.38 (2.5-7.1) | 22.4 |
| Omitting Lasnibat et al., 2017 | 4.82 (2.6-7.1) | 21.8 |
| Omitting Amor et al., 2020 | 4.69 (2.7-7.5) | 4.2 |
| Omitting Olmi et al., 2020 | 4.99 (2.3-7.2) | 0.0 |
|  |  |  |
| **Pooled estimate** | **2.9 (0.0-8.3)** | **76.5** |
|  |  |  |
| **Overall complications** | Proportion (95% CI) | I^2^ |
| Omitting Silva et al., 2015 | 11.5 (8.6-15.5) | 34.1 |
| Omitting Moon et al., 2016 | 9.9 (6.4-15.2) | 53.7 |
| Omitting Nocca et al., 2016 | 8.5 (5.5-12.8) | 40.2 |
| Omitting Lasnibat et al., 2017 | 9.2 (5.8-14.3) | 56.4 |
| Omitting Amor et al., 2020 | 10.8 (7.6-15.1) | 38.8 |
| Omitting Olmi et al., 2020 | 8.3 (4.7-14.3) | 45.1 |
|  |  |  |
| **Pooled estimate** | **9.8 (5.7-13.4)** | **37.6** |

**Supplementary Table 1**. One-leave out sensitivity analysis for primary outcomes. Results are expressed in pooled proportions and 95% Confidence Intervals (95%CI). I^2^: heterogeneity.
